# The tongue may not move, but the voice will: preoperative AI voice cloning for identity preservation in major glossectomy — a prospective feasibility study

**DOI:** 10.3389/fonc.2026.1915964

**Published:** 2026-09-21

**Authors:** Prajwal S. Dange, Karthik N. Rao, Kumareshwar TV, Teertha Shetty, Radhika Kapahtia, Krishna Chaitanya, Sreeram MP

**Affiliations:** 1 Department of Head and Neck Surgical Oncology, Sri Shankara Cancer Hospital and Research Centre, Bengaluru, India; 2 Department of Head and Neck Surgical Oncology, Speech and swallow division, Sri Shankara Cancer Hospital and Research Centre, Bengaluru, India; 3 Department of Plastic and Reconstructive Surgery, Sri Shankara Cancer Hospital and Research Centre, Bengaluru, India; 4 Department of Data Sciences and Artificial intelligence, Sri Shankara Cancer Hospital and Research Centre, Bengaluru, India

**Keywords:** major glossectomy, multilingual speech synthesis, speaker similarity, tongue neoplasms, voice banking

## Abstract

**Background:**

Major glossectomy frequently results in profound speech impairment. We investigated whether a patient’s voice could be captured preoperatively and reconstructed using artificial intelligence voice cloning for potential future use as a personalized assistive communication voice during postoperative recovery.

**Methods:**

In this prospective single-centre feasibility study, adult patients with treatment-naïve carcinoma of the tongue planned for major glossectomy underwent a single approximately 30–60 second preoperative voice recording in a sound-treated room. Voices were cloned offline using the IndicF5 flow-matching text-to-speech model, generating a standardized reading-passage output and a patient-transcript output for each participant. Speaker fidelity was assessed using cosine similarity across three independent speaker-verification encoders, calibrated against each patient’s intra-speaker and cohort impostor distributions, alongside mel-cepstral distortion and prosodic feature analysis. Subjective acceptance was assessed using the Personalised Synthetic Voice Acceptance Questionnaire across six domains, completed by patients and family members.

**Results:**

Of 34 enrolled patients, 26 formed the final cohort across four languages. Mean cosine similarity was 0.933 (Resemblyzer), 0.969 (WavLM), and 0.806 (ECAPA-TDNN), with clone scores generally falling within patient-specific intra-speaker distributions and above cohort impostor distributions. Mean mel-cepstral distortion was 7.87 dB. Fundamental frequency and other prosodic measures showed broad cohort-level correspondence with variable individual deviations. Identity-and-Ownership and Authenticity-and-Trust domains scored highest among both patients and family or attenders, while naturalness and emotional acceptance were rated more conservatively.

**Conclusion:**

These findings demonstrate the feasibility of generating personalised synthetic voices from brief preoperative clinic-grade recordings, with objective speaker similarity and preliminary patient- and family-reported acceptance. The findings support further evaluation of these voices as potential assistive communication tools during postoperative recovery; however, postoperative communication performance and clinical benefit were not evaluated in the present study.

## Introduction

Carcinoma of the tongue remains one of the most common malignancies of the oral cavity in South Asia, where smokeless tobacco use, areca-nut consumption, and related exposures continue to drive disease incidence (1–4). Patients presenting with locally advanced disease frequently require major glossectomy as part of curative management (5). While advances in surgical technique, microvascular reconstruction, and adjuvant therapy have meaningfully improved survival over the past two decades, the functional consequences of extensive tongue resection remain substantial, and the impaired speech is usually where this burden is felt most (3).

Speech impairment is among the most disabling sequelae of major glossectomy. Even with optimal reconstruction, many patients are left with persistent reductions in intelligibility that compromise everyday communication (6). These deficits are rarely confined to function alone; they extend into social participation, emotional wellbeing, and overall quality of life. Qualitative work in this area has consistently shown that patients do not experience loss of voice as a purely mechanical impairment. It is more often described in terms of identity, as though something central to the self has been altered along with the ability to speak (7–9).

Prehabilitation has gained traction in head and neck oncology, with most efforts directed at optimizing nutrition, swallowing function, and speech therapy ahead of treatment (10). One area that has received comparatively little to no attention is the preservation of a patient’s own voice for use during postoperative recovery. Voice banking, the practice of recording and storing an individual’s voice in anticipation of its loss, is well established in conditions such as amyotrophic lateral sclerosis and other progressive speech disorders (11–14). Its clinical use has been limited, however, by the practical demands of conventional approaches, which typically require several hours of high-quality recording and are difficult to incorporate into routine preoperative workflows (15).

Recent developments in Artificial intelligence based neural text-to-speech technology have begun to address this limitation (16). Zero-shot voice cloning systems are now capable of producing speaker-faithful synthetic speech from a brief reference recording, while still retaining the key vocal characteristics of the speaker (17). This makes it feasible, for the first time, to generate a personalized synthetic voice from audio obtained during a routine preoperative visit, without requiring extended dedicated recording sessions. Objective acoustic measures can quantify how closely a cloned voice resembles the original, but acoustic similarity alone does not determine clinical success.

What ultimately matters is whether the patient and their family perceive the synthesized voice as representing the patient’s own identity, rather than as a generic or unfamiliar approximation.

Evidence on this question remains limited, particularly in patients undergoing major glossectomy for oral cavity cancer. We therefore conducted a prospective, single-center feasibility study to evaluate whether adult patients with carcinoma of the tongue, along with their family members, perceived a preoperatively generated AI-cloned voice as representing the patient’s vocal identity. Acceptance was assessed across six predefined domains and examined alongside objective measures of speaker similarity, spectral fidelity, and prosodic preservation. The study was not designed to evaluate postoperative speech intelligibility, restoration of articulation, communication effectiveness, or quality-of-life benefit.

## Materials and methods

### Study design and setting

This was a prospective feasibility study conducted at Sri Shankara Cancer Hospital and Research Centre, Bengaluru. We enrolled consecutive adult patients with biopsy-proven carcinoma of the tongue who were planned for primary surgical resection from April to May 2026. Institutional Ethics Committee clearance was sought for the conduct of this study prior to patient enrolment in accordance with the 2024 revision of the Declaration of Helsinki (vide letter no SSCHRC/IEC29/269 dated 28.3.2026). Written informed consent was obtained individually from every participant, with the study procedures, the nature of voice recording and cloning, and the associated risks explicitly explained beforehand; consent specifically covered voice recording, voice cloning, and the use of cloned outputs for research and clinical communication, given the novel nature of this intervention.

### Patient selection

Eligibility was restricted to patients planned for a major glossectomy, whether hemi-, sub-total, or total, who had treatment-naïve disease with no prior surgery, radiotherapy, or chemotherapy directed at the index tumor. We required conversational speech intelligibility judged adequate at clinical interview, using the AYJNIHH 7-point Speech Intelligibility Rating Scale (18) as a reference standard, and patients had to be able to read aloud comfortably in their preferred vernacular language. Normal conversational hearing without a hearing aid was also required, as was the absence of a nasogastric tube or tracheostomy tube at the time of recording, since either would distort the reference audio. Patients who did not meet these criteria were excluded, along with anyone whose source recording proved acoustically insufficient to serve as a reliable reference clip.

### Voice recording and pre-processing

The voice-cloning pipeline began with a single preoperative recording obtained inside a sound-treated pure-tone audiometry room, using an Apple iPhone 15 Pro integrated microphone at AAC encoded audio (16-bit equivalent acquisition using the native iPhone Voice Memos application). Patients were simply asked to introduce themselves, speaking about their daily life, occupation, family, and presenting complaint in whichever language they were most comfortable with, and recordings ran between 30 and 60 seconds. Files were transferred to a secure local workstation immediately afterward, with patient identifiers stripped at the point of transfer and replaced with the medical record document number; no audio left our internal systems or was sent to any external service at any stage.

### Voice-cloning pipeline

For the cloning step itself, we used the open-source IndicF5 flow-matching text-to-speech model from AI4Bharat (19). The model was pinned to a fixed release-tag commit to ensure reproducibility (20). For Indian-English speakers, the target text was routed through IndicF5 using Devanagari phonetic transliteration, as pilot testing showed that the standard English F5-TTS Base checkpoint did not adequately preserve the Indian-English accent. The entire pipeline ran offline on a CPU-only workstation, with software versions pinned throughout, including PyTorch 2.5.1 (21), Hugging Face Transformers 4.46.3 (22), OpenAI Whisper (23), and SpeechBrain 1.1 (24). Because the F5-TTS family generates speech conditioned on a paired reference audio and reference transcript, we extracted a 10 to 12 second denoised reference clip from the start of each recording (25). Before cloning, each file underwent high-pass filtering at 80 Hz and low-pass filtering at 8 kHz, adaptive frequency-domain denoising, and resampling to mono 24 kHz.

Reference transcripts were prepared directly by the treating clinician team for Kannada, Tamil, and Hindi speakers, while English transcripts were generated automatically using Whisper and then verified by the clinician.

Two cloned outputs were produced for each participant. Output A consisted of a standardised reading passage that was not derived from the participant’s recorded speech and was used to provide a more consistent cross-participant assessment. Output B was generated from the participant’s own preoperative transcript and therefore provided an identical-content comparison with the source recording. Because Output B shared linguistic and phonetic content with the reference material, its similarity measures were considered potentially more favourable and were interpreted separately from the standardised Output A assessment. Output B was included to assess within-participant acoustic correspondence under content-matched conditions and was not considered an independent test of generalisation to unseen linguistic content. The two outputs therefore served complementary purposes: Output A provided a standardised assessment condition, whereas Output B provided a content-matched within-participant comparison.

### Objective acoustic evaluation

Objective evaluation of speaker identity preservation relied on cosine similarity between speaker’s original recording and the cloned output (26). Rather than depend on a single encoder, we used three independently trained speaker-verification systems to check cross-encoder agreement: the generalized end-to-end d-vector encoder of Wan and colleagues (27), implemented through the Resemblyzer package (28); the WavLM-base-plus-sv speaker-verification head described by Chen and colleagues (29); and the ECAPA-TDNN encoder of Desplanques and colleagues (30), in its SpeechBrain VoxCeleb-trained form (31).

Since absolute cosine similarity values vary by encoder and don’t transfer meaningfully across studies, we adopted a cohort-internal calibration strategy instead of relying on fixed thresholds (32). For each patient, we built a genuine distribution from pairwise intra-speaker cosine similarities across overlapping sliding windows of their own original recording, and an impostor distribution from inter-speaker cosine similarities comparing that patient’s centered reference window against duration-matched windows from every other patient’s original recording. The clone score itself was taken as the mean cosine similarity between the cloned output and three duration-matched segments of the patient’s original audio.

These measures were selected because they evaluate complementary aspects of synthetic voice quality. Speaker encoder cosine similarity provides an embedding-based estimate of speaker identity preservation independent of linguistic content. Mel-cepstral distortion quantifies spectral similarity between original and synthesized speech and is widely used as an objective measure of speech synthesis quality. Fundamental frequency and its variability assess preservation of pitch characteristics, whereas jitter, shimmer, and harmonics-to-noise ratio evaluate voice quality and micro-instability. Collectively, these measures provide a multidimensional assessment of speaker fidelity that captures complementary aspects of similarity rather than relying on a single acoustic metric.

Spectral fidelity was assessed separately using dynamic-time-warping-aligned mel-cepstral distortion (33), computed with the pymcd package using 13th-order mel-cepstral coefficients (34), and prosodic features, mean fundamental frequency, fundamental frequency standard deviation, local jitter, local shimmer, and harmonics-to-noise ratio, were extracted in Praat 6.4 through the Parselmouth Python bindings (35), with identical analysis settings applied to both the original and the cloned audio (pitch floor 75 Hz, ceiling 500 Hz, time step 0.01 seconds).

### Subjective acceptance assessment

Subjective acceptance was captured using a custom rubric we developed for this study, the Personalised Synthetic Voice Acceptance Questionnaire (PSV-AQ) (**Table 1**). It exists in two parallel versions, one for patient self-report and one for a family member or attender, and both are organized around six perceptual domains: naturalness, similarity, identity and ownership, emotional acceptance, authenticity and trust, and intention or endorsement. The PSV-AQ was administered during the first postoperative follow-up after patients had listened to their cloned voice samples. Both patients and accompanying family members independently completed their respective questionnaires immediately following playback of the synthesized recordings.

**Table 1:** Personalised Synthetic Voice Acceptance Questionnaire (PSV-AQ).

Domain	Item no.	Questionnaire item	Reverse scored
Naturalness (MOS-N anchor)	1	The cloned voice sounds like a person speaking, not a machine.	No
	2	The cloned voice has natural prosody and intonation.	No
	3	The cloned voice sounds robotic or unnatural.	Yes
Similarity (Sim-MOS anchor)	4	The cloned voice sounds like the patient’s voice.	No
	5	The cloned voice has the same accent and articulation as the patient.	No
	6	The cloned voice does not match the patient’s voice.	Yes
Identity and ownership	7	I would recognise this voice as the patient’s voice.	No
	8	If I heard this voice on a phone call, I would believe it was the patient.	No
	9	The cloned voice belongs to the patient.	No
Emotional acceptance	10	I feel comfortable hearing the cloned voice.	No
	11	Hearing the cloned voice does not cause distress.	No
	12	The cloned voice is distressing or uncomfortable to hear.	Yes
Authenticity and trust	13	The cloned voice is an authentic representation of the patient.	No
	14	I would trust this voice to communicate the patient’s words on their behalf.	No
	15	The cloned voice feels artificial or untrustworthy.	Yes
Intention and endorsement	16	I would want to use this cloned voice in the postoperative period.	No
	17	I would recommend this approach to other patients with similar needs.	No
	18	I would endorse using the cloned voice for everyday communication.	No
Scoring	–	Reverse-scored items are recoded (6 − raw score). Domain scores are calculated as the mean of the item scores within each domain and reported on a 1–5 scale.	–
Versions	–	Two parallel versions are used: (a) Patient self-report version, in which the patient rates the cloned voice from their own perspective; and (b) Family/attender version, in which a family member or attender who has known the patient for ≥3 months rates the same voice samples. Both versions contain identical items and use identical scoring.	–

All items are scored on a 5-point Likert scale (1 = strongly disagree, 5 = strongly agree). Reverse-scored items are recoded (6 − raw score) before calculating domain scores. Domain scores are reported as the mean of the recoded item scores (range 1–5). The same questionnaire is administered to patients and to family members/attenders who have known the patient for at least 3 months.

The naturalness domain uses the Mean Opinion Score Absolute Category Rating anchor from ITU-T Recommendation P.800.1, and the similarity domain uses the corresponding Similarity Mean Opinion Score anchor (36); items for the remaining four domains were derived from the existing voice-banking acceptance literature. It is worth being explicit that this questionnaire has not undergone formal psychometric validation. Each item was rated on a 1 to 5 scale, with reverse-scored items recoded prior to analysis, and domain-level means were calculated separately for the patient and family or attender responses.

### Data compilation and statistical analysis

All acoustic measurements, questionnaire responses, and clinical variables were compiled into a structured Microsoft Excel spreadsheet, and statistical analysis was performed using R version 4.5.2 (R Foundation for Statistical Computing, Vienna, Austria). Descriptive data are presented as mean with standard deviation or median with range, as appropriate to the distribution. Original and cloned recordings were compared within participants for descriptive assessment of prosodic and acoustic measures. As this was a prospective feasibility study, analyses were primarily descriptive and intended to characterize acoustic fidelity rather than formally test predefined hypotheses. Objective similarity measures were interpreted relative to patient-specific genuine and impostor distributions rather than universal thresholds because cosine similarity values are encoder-dependent.

## Results

### Cohort and voice synthesis outcomes

Thirty-four patients consented to participate and underwent preoperative voice recording. Eight participants (23.5%) were excluded because their recordings did not meet the requirements for use as reliable reference clips, leaving a final cohort of 26 patients (76.5% successful acquisition). The excluded recordings were not included in subsequent synthesis or acoustic analyses. This exclusion rate was considered part of the feasibility assessment of the proposed workflow. Demographic, oncological, and procedural characteristics are summarized in **Table 2**.

**Table 2:** Per-patient demographic, oncological, and procedural characteristics.

PID	Age	Sex	cTN	Stage	Surgery	AYJ pre-op	Adj Tx	Rec dur (s)	Language
P01	72	M	T4 N1	IVA	Total glossectomy + bilateral ND + flap	3	Adj CT-RT	44	Kannada
P02	45	M	T4 N1	IVA	Total glossectomy + bilateral ND + flap	2	Adj CT-RT	37	Kannada
P03	74	F	T2 N1	III	Hemiglossectomy + ND + flap	3		41	Hindi
P04	76	F	T3 N1	III	Near-total glossectomy + bilateral ND + flap	2	Adj RT	30	Kannada
P05	59	F	T4 N1	IVA	Total glossectomy + bilateral ND + flap	1	Adj CT-RT	55	Kannada
P06	62	M	T1 N1	III	Hemiglossectomy + ND + flap	2		46	Kannada
P07	53	M	T2 N1	III	Hemiglossectomy + ND + flap	3		44	Kannada
P08	48	F	T2 N0	II	Hemiglossectomy + ND + flap	1		54	Kannada
P09	62	M	T2 N1	III	Hemiglossectomy + ND + flap	1		33	Kannada
P10	62	F	T3 N0	III	Hemiglossectomy + ND + flap	1	Adj RT	38	Kannada
P11	57	M	T2 N0	II	Hemiglossectomy + ND + flap	1		47	Kannada
P12	68	M	T1 N1	III	Hemiglossectomy + ND + flap	2		40	Tamil
P13	52	M	T3 N1	III	Near-total glossectomy + bilateral ND + flap	2	Adj RT	59	Kannada
P14	76	M	T1 N1	III	Hemiglossectomy + ND + flap	3		44	Indian English
P15	38	M	T1 N1	III	Hemiglossectomy + ND + flap	2		34	Indian English
P16	81	M	T1 N0	I	Hemiglossectomy + ND + flap	1	None	42	Indian English
P17	67	M	T1 N1	III	Hemiglossectomy + ND + flap	1		41	Indian English
P18	80	M	T3 N1	III	Hemiglossectomy + ND + flap	2	Adj RT	60	Kannada
P19	40	F	T2 N1	III	Hemiglossectomy + ND + flap	1		76	Tamil
P20	56	M	T1 N1	III	Hemiglossectomy + ND + flap	1		55	Indian English
P21	48	M	T1 N0	I	Hemiglossectomy + ND + flap	3		45	Kannada
P22	61	F	T2 N1	III	Hemiglossectomy + ND + flap	1		77	Kannada
P23	53	F	T3 N1	III	Hemiglossectomy + ND + flap	3	Adj RT	33	Indian English
P24	66	M	T3 N0	III	Hemiglossectomy + ND + flap	2	Adj RT	30	Indian English
P25	76	M	T1 N1	III	Hemiglossectomy + ND + flap	2		51	Indian English
P26	51	M	T1 N1	III	Hemiglossectomy + ND + flap	3		31	Kannada

PID, patient anonymisation code; Ctn, clinical TN stage; Stage, composite AJCC 8th edition stage; AYJ pre-op, pre-operative AYJNIHH 7-point Speech Intelligibility Rating Scale score; Adj Tx, planned adjuvant treatment; Rec dur (s), preoperative recording duration in seconds. ND, neck dissection.

The cohort reflected the linguistic diversity we routinely encounter in this region, with four spoken languages represented: Kannada (n = 15), Indian English (n = 8), Tamil (n = 2), and Hindi (n = 1). The mean duration of the preoperative voice recording was 41 seconds (range, 29–64 seconds), well within what we had anticipated would be feasible to capture during a routine clinic visit. Two synthetic voice files were generated for each patient, yielding 52 outputs in total. Output A, the standardized reading passage, had a mean duration of 38 seconds, while Output B, synthesized from the patient’s own transcript, averaged 34 seconds. Both outputs were generated successfully in every patient, with no failures in the pipeline at this stage.

### Objective acoustic similarity

Across all three speaker-verification encoders, cloned voices demonstrated speaker identity preservation. Mean cosine similarity was 0.933 using Resemblyzer, 0.969 using WavLM, and 0.806 using ECAPA-TDNN. Clone scores generally approximated each patient’s intra-speaker similarity distribution while remaining above the cohort impostor distribution (**Table 3**). The distribution of clone scores relative to the intra-speaker and impostor benchmarks for each encoder is shown in **Figures 1**–**3**.

**Table 3:** Per-patient objective acoustic similarity and prosodic preservation.

PID	Resemblyzer	WavLM	ECAPA	MCD (dB)	F0 orig	F0 clone	Jitter o/c	Shimmer o/c
P01	0.918	0.940	0.801	10.30	163.0	125.7	0.041/0.027	0.193/0.140
P02	0.834	0.958	0.500	6.49	189.5	185.1	0.013/0.020	0.059/0.083
P03	0.969	0.985	0.864	8.11	130.1	121.7	0.025/0.023	0.111/0.098
P04	0.947	0.970	0.790	7.72	178.2	181.5	0.021/0.020	0.096/0.095
P05	0.921	0.967	0.801	6.86	173.6	189.0	0.023/0.016	0.104/0.094
P06	0.948	0.961	0.854	9.40	123.4	118.3	0.021/0.022	0.095/0.091
P07	0.918	0.957	0.825	8.49	120.9	113.0	0.053/0.026	0.185/0.120
P08	0.934	0.952	0.817	7.50	169.0	179.9	0.021/0.013	0.092/0.077
P09	0.935	0.974	0.854	10.04	151.4	148.7	0.024/0.022	0.103/0.100
P10	0.919	0.984	0.849	9.92	197.8	193.3	0.029/0.019	0.159/0.112
P11	0.946	0.964	0.770	7.42	142.4	144.0	0.018/0.016	0.095/0.078
P12	0.950	0.982	0.849	9.94	116.8	114.4	0.024/0.020	0.110/0.086
P13	0.950	0.968	0.856	7.28	89.1	96.7	0.026/0.019	0.084/0.073
P14	0.960	0.965	0.870	7.63	128.9	132.3	0.036/0.024	0.101/0.097
P15	0.921	0.981	0.812	6.29	122.7	123.4	0.015/0.016	0.109/0.083
P16	0.929	0.962	0.734	6.32	157.3	158.6	0.019/0.018	0.093/0.104
P17	0.971	0.983	0.843	7.86	95.3	95.9	0.027/0.026	0.129/0.118
P18	0.940	0.961	0.874	6.97	159.7	154.2	0.030/0.022	0.142/0.108
P19	0.927	0.978	0.860	6.99	172.7	170.5	0.024/0.012	0.095/0.067
P20	0.964	0.977	0.880	6.84	126.3	120.8	0.027/0.024	0.119/0.087
P21	0.901	0.967	0.855	7.21	108.9	109.8	0.039/0.027	0.143/0.108
P22	0.915	0.980	0.774	6.24	149.4	148.5	0.029/0.030	0.116/0.101
P23	0.938	0.984	0.829	6.48	199.5	192.6	0.021/0.019	0.084/0.092
P24	0.939	0.968	0.771	7.89	111.9	109.3	0.017/0.020	0.087/0.097
P25	0.927	0.957	0.675	10.30	100.7	97.5	0.042/0.025	0.145/0.111
P26	0.934	0.978	0.752	8.22	110.0	106.6	0.034/0.029	0.175/0.136

Resemblyzer, WavLM, ECAPA = speaker encoder cosine similarity between the original recording and the patient’s two cloned outputs (passage + own transcript), averaged. MCD, dynamic-time-warping-aligned mel-cepstral distortion, dB. F0 orig/F0 clone = mean fundamental frequency in the original and clone, Hz. Jitter o/c and Shimmer o/c are local Praat measures for original/clone.

**Figure 1:**
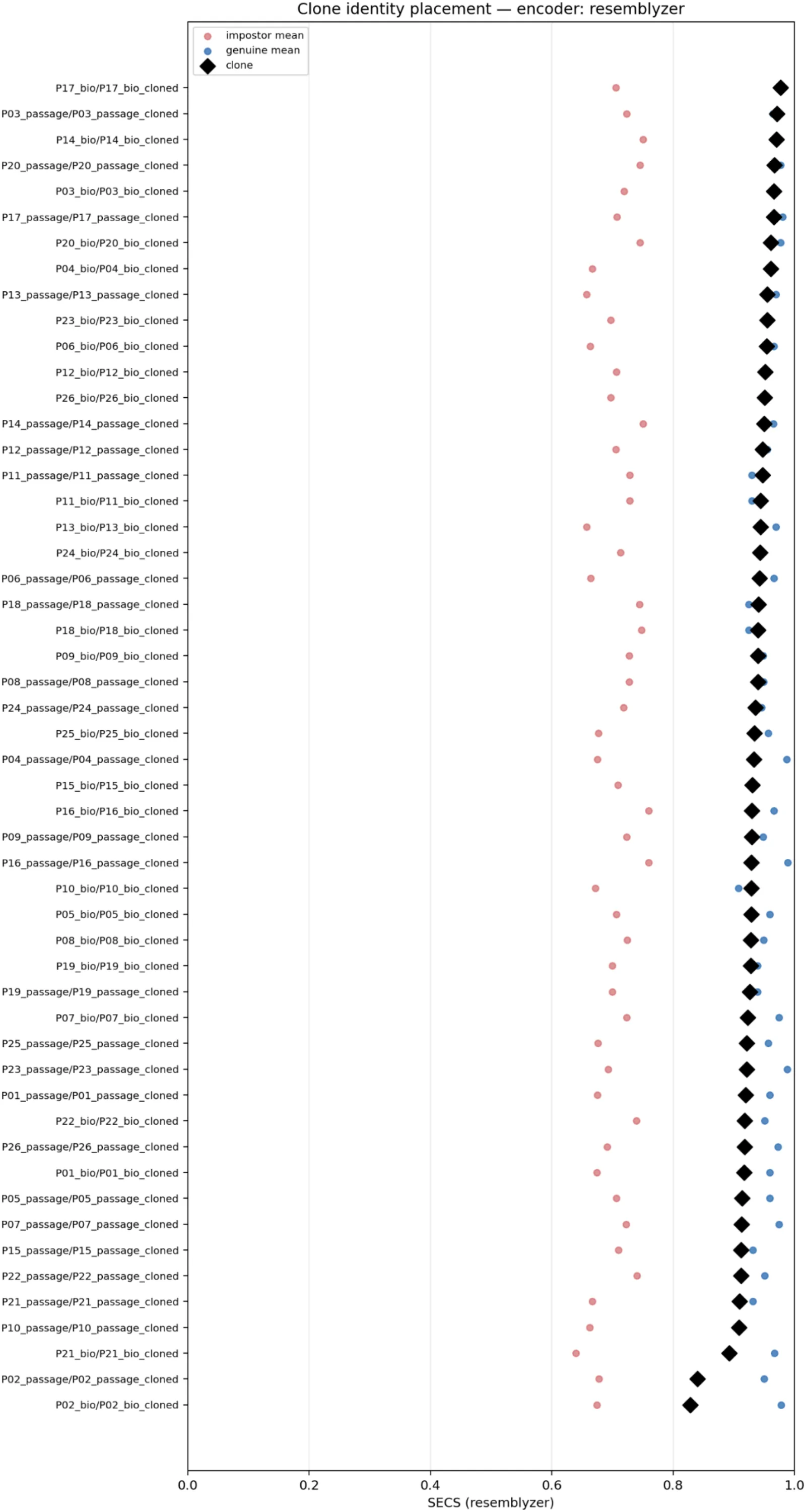
Per-patient speaker encoder cosine similarity placement using the Resemblyzer (generalized end-to-end d-vector) encoder. Each row is one cloned output. The black diamond is the clone cosine similarity, the blue dot is the mean of the patient’s intra-speaker (genuine) distribution, and the red dot is the mean of the impostor distribution. Patient codes P01 to P26 are assigned in enrolment order.

**Figure 2:**
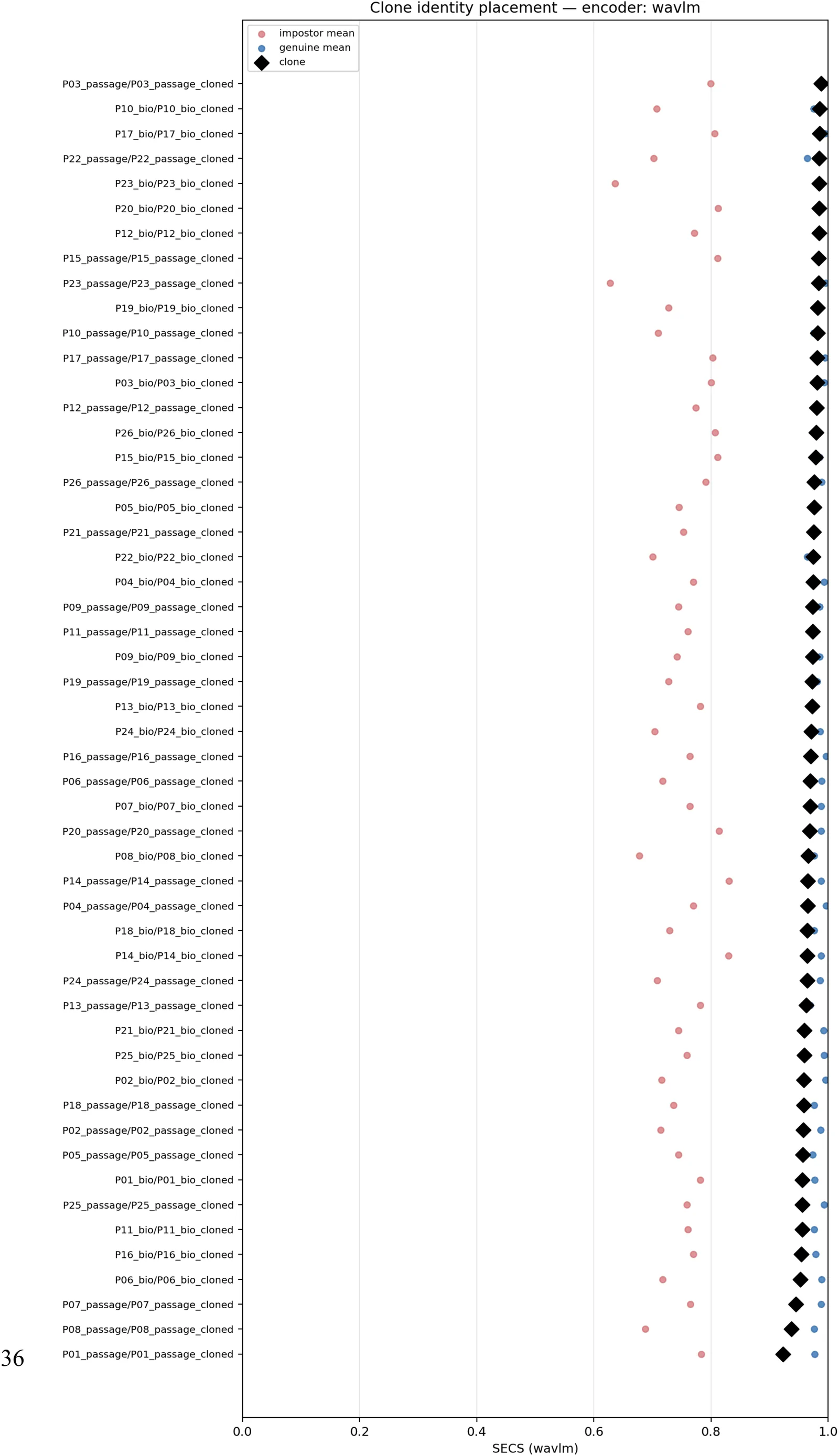
Per-patient cosine similarity placement using the WavLM-base-plus-sv encoder. Each row is one cloned output. The black diamond is the clone cosine similarity, the blue dot is the mean of the patient’s intra-speaker (genuine) distribution, and the red dot is the mean of the impostor distribution. Patient codes P01 to P26 are assigned in enrolment order.

**Figure 3:**
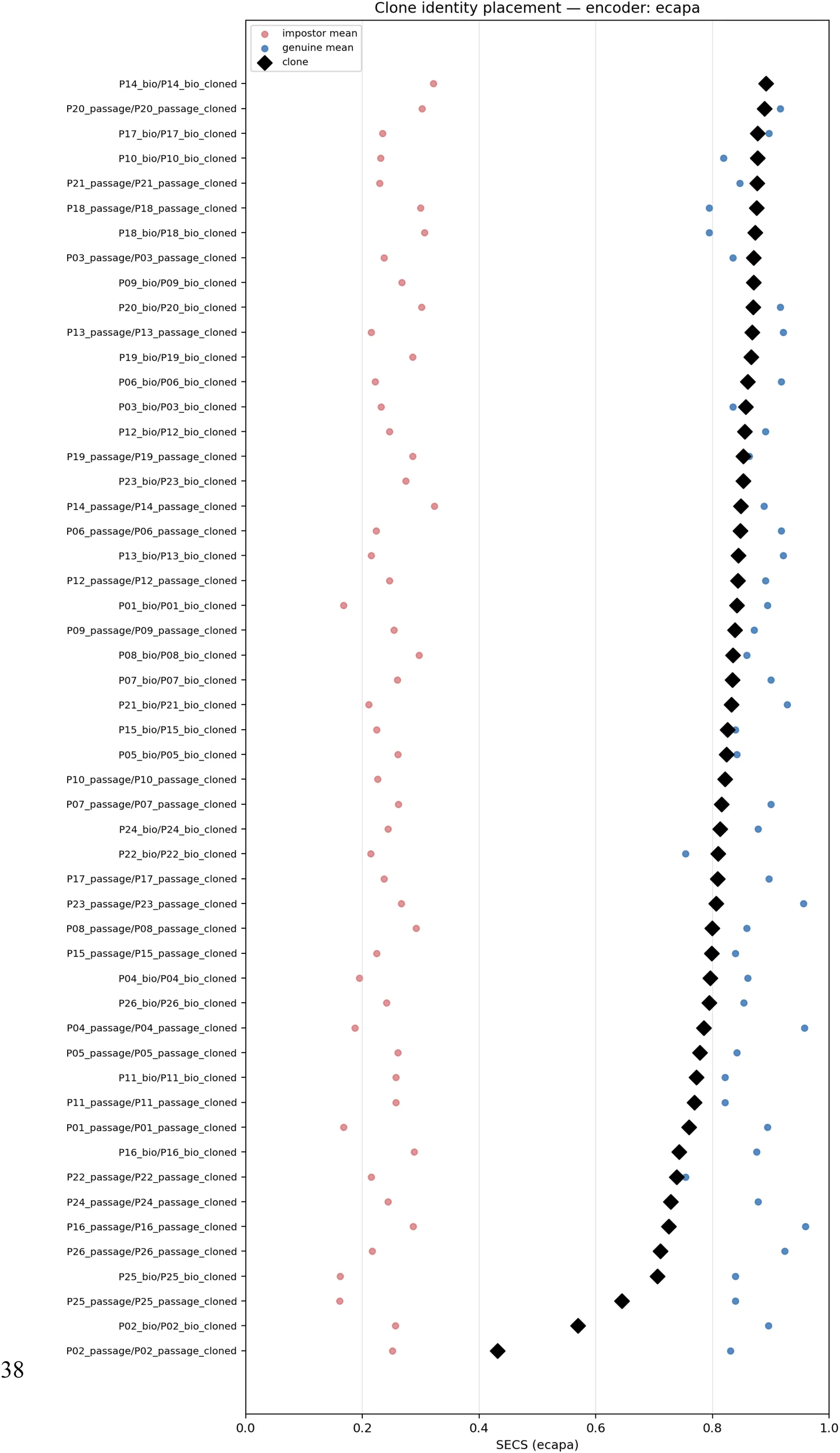
Per-patient cosine similarity placement using the ECAPA-TDNN encoder. Each row is one cloned output. The black diamond is the clone cosine similarity, the blue dot is the mean of the patient’s intra-speaker (genuine) distribution, and the red dot is the mean of the impostor distribution. Patient codes P01 to P26 are assigned in enrolment order.

Outputs generated from the patient’s own transcript (Output B) showed marginally higher cosine similarity than the standardized reading-passage outputs (Output A), which is not entirely surprising given that Output B shares identical phonetic content with the original recording, whereas Output A does not. Spectral correspondence was observed between the original and synthesized recordings. Mean dynamic-time-warping-aligned mel-cepstral distortion was 7.87 dB (range 4.21–11.86 dB), indicating measurable spectral similarity within the analysis framework used. Prosodic analysis showed broad correspondence between original and cloned recordings (**Table 3**). Mean fundamental frequency showed a small cohort-level difference, although individual participants demonstrated variable deviations. F0 variability showed similar overall distributions, with greater individual variation in some participants. Jitter, shimmer, and harmonics-to-noise ratio also differed between original and synthesized recordings. Representative examples from three patients (P01, P02, and P03) are presented in **Figure 4** to illustrate the observed correspondence in mel-spectrograms and fundamental-frequency contours.

**Figure 4:**
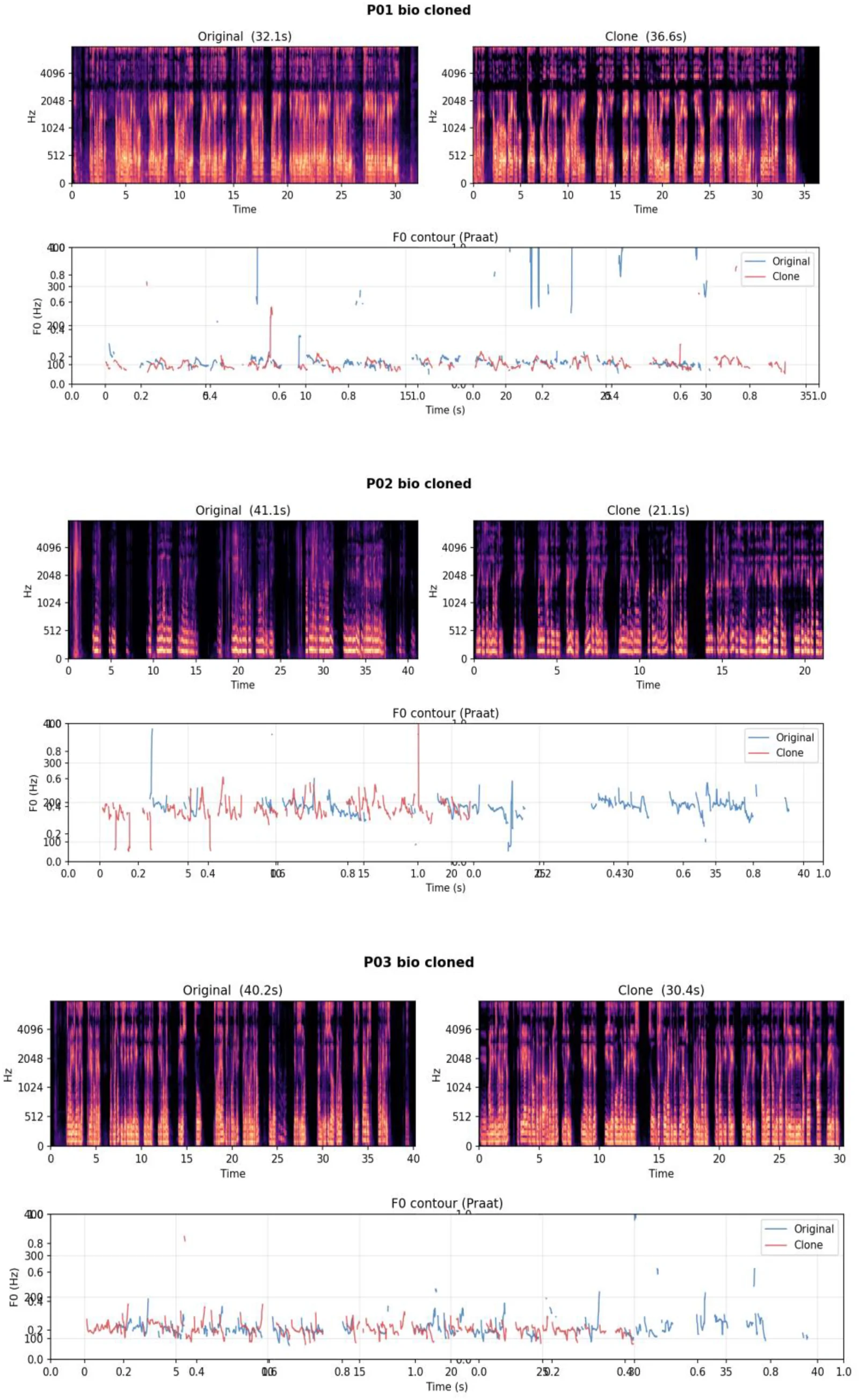
Per-patient acoustic comparison panels for three representative patients. For each patient the top row shows mel-spectrograms of the original (left) and the clone (right) up to 8 kHz, and the bottom panel overlays fundamental frequency contours computed in Praat (original in blue, clone in red).

### Subjective cceptance

Patient- and family-reported PSV-AQ domain scores are summarized in **Table 4** and illustrated in **Figure 5**, with patient and family or attender responses presented separately to reflect related but distinct perspectives on perceived identity and acceptance of the cloned voice.

**Table 4:** Per-patient personalised synthetic voice acceptance questionnaire scores (patient self-report).

Patient	Naturalness	Similarity	Identity	Emotional	Authenticity	Endorsement
P01	4	3	5	2	5	4
P02	4	4	5	2	5	2
P03	4	4	4	2	4	4
P04	5	3	3	3	4	2
P05	4	4	5	3	4	4
P06	4	4	4	3	4	3
P07	4	4	4	2	5	4
P08	4	5	5	2	4	4
P09	3	3	5	3	5	3
P10	5	3	4	3	5	3
P11	4	2	5	3	4	3
P12	4	4	4	2	5	5
P13	4	4	4	3	5	4
P14	4	3	5	3	4	3
P15	3	4	5	3	5	3
P16	3	4	5	3	4	4
P17	4	4	4	2	5	4
P18	5	4	5	3	5	3
P19	3	3	5	1	4	4
P20	3	3	5	3	4	4
P21	4	4	4	2	3	3
P22	5	3	5	4	5	4
P23	4	3	5	2	5	3
P24	5	3	4	4	4	4
P25	4	3	4	2	5	3
P26	3	4	4	1	4	4

Each cell is the patient’s mean domain score on the 1 to 5 integer scale. Naturalness was anchored to ITU-T P.800.1 MOS-N; Similarity was anchored to Sim-MOS; the remaining four domains were scored on the Personalised Synthetic Voice Acceptance Questionnaire 1 to 5 scale. Family or attender ratings are provided in **Supplementary File 2**.

**Figure 5:**
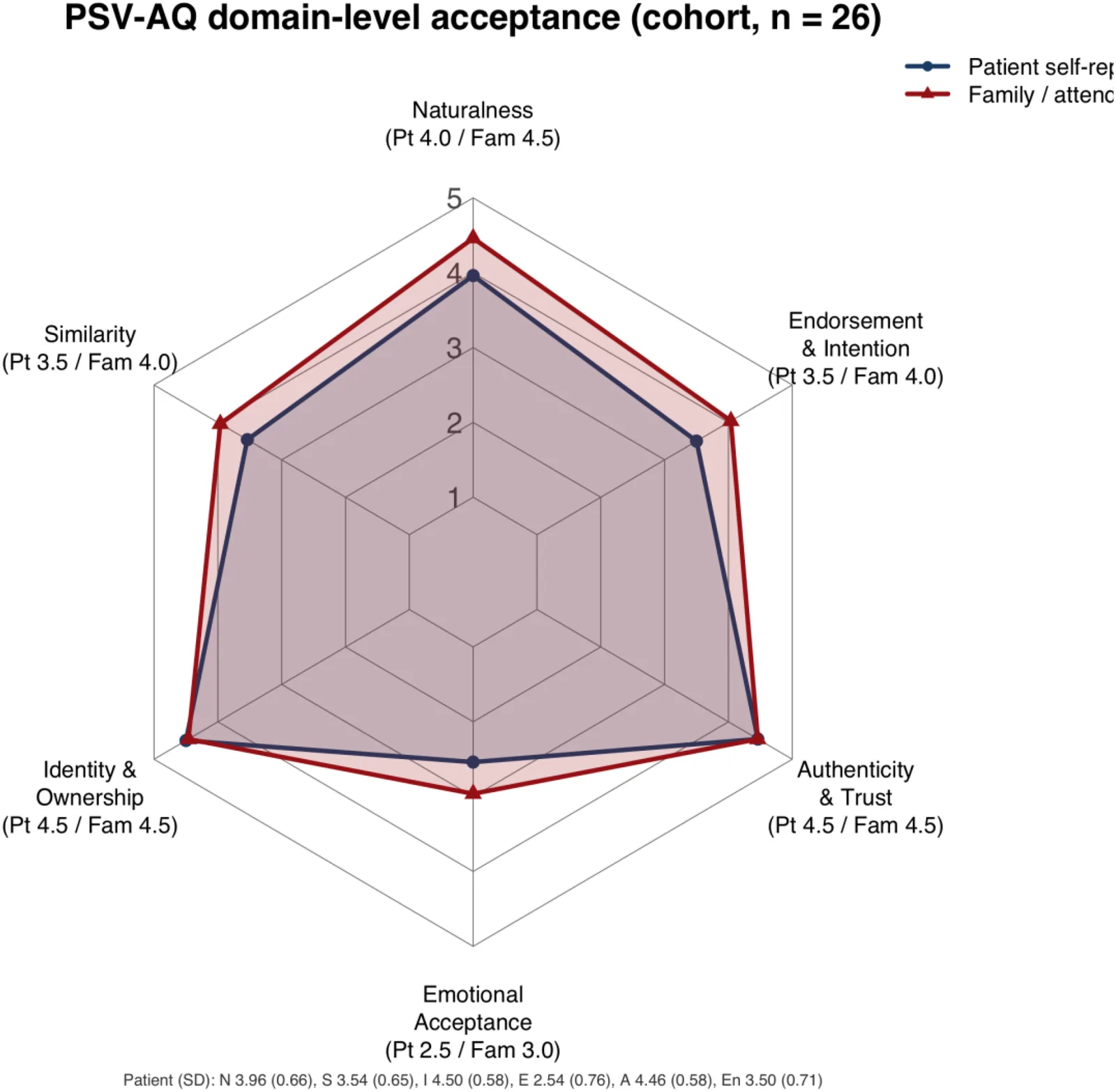
Personalised Synthetic Voice Acceptance Questionnaire domain-level acceptance ratings, patient self-report versus family or attender. Six-axis radar plot of the Personalised Synthetic Voice Acceptance Questionnaire cohort-mean domain scores on the 1 to 5 scale (n = 26), showing patient self-report and family or attender ratings. Identity and Ownership and Authenticity and Trust showed the highest domain scores in both groups, while the remaining domains showed somewhat higher ratings among family or attenders. The figure is descriptive and does not imply statistical significance between respondent groups.

## Discussion

### Principal findings

Across three independently trained speaker-verification encoders, the cloned outputs in this cohort generally showed speaker-similarity scores within the range of each patient’s intra-speaker distribution and above the cohort impostor distribution. Mean fundamental frequency and its variability showed broad correspondence between original and cloned recordings, although individual deviations were observed. The cohort-level DTW-aligned mel-cepstral distortion was 7.87 dB. The pipeline operated entirely offline on a single clinician’s workstation and generated outputs in four represented languages. These findings support the technical feasibility of personalised synthetic voice generation from brief clinic-grade recordings, while language-specific and patient-level performance require further evaluation.

### Comparison with the voice-banking literature

Voice banking has been studied most extensively in amyotrophic lateral sclerosis, a population in which the trajectory of voice loss is gradual and permits extended preparation (11–13). Hyppa-Martin and colleagues compared concatenative voice-banking with neural speaker-adapted synthesis in voices banked by individuals with ALS and reported that the neural output was rated as more intelligible, more natural, and more similar to the original speaker than the concatenative output (37). Costello and Smith, describing the Boston Children’s Hospital workflow of pairing message banking with voice banking, emphasized that the intervention is meaningful only when carried out with sufficient lead time before vocal decline (38). The head and neck cancer population differs from these cohorts in two clinically decisive respects: the enrolment window is measured in hours to days rather than weeks to months, since surgery is scheduled and the resulting voice loss is abrupt; and the source material is clinic-grade smartphone audio rather than studio enrolment. Zero-shot text-to-speech is the architecture suited to these constraints, as it removes the dedicated speaker-enrolment session that has historically been the rate-limiting step in voice banking (39). The brain-to-voice neuroprosthesis work of Littlejohn and colleagues, which restores naturalistic communication in severely anarthric speakers through an implantable device, occupies a different point on this spectrum; while not applicable to our surgical population, it illustrates the pace at which personalized speech-synthesis capability is advancing (40). The present findings suggest that a prolonged dedicated speaker-enrolment session may not be necessary for generating a personalised synthetic voice in this feasibility setting. Whether this approach can be incorporated reliably into routine clinical workflows requires evaluation in larger, multicentre cohorts. To our knowledge, prospective evaluation of personalised synthetic voice generation in patients undergoing major glossectomy remains limited, particularly in multilingual Indian clinical settings.

### Speaker similarity and acoustic fidelity in context

In the zero-shot text-to-speech literature, speaker similarity is reported either as cosine similarity between speaker-verification embeddings or as a listener-rated Similarity Mean Opinion Score (41). Absolute cosine similarity values are encoder-dependent and not directly portable between studies (42); Casanova and colleagues, in their work on YourTTS, demonstrated that an identical set of cloned voices can shift in absolute similarity score by a margin larger than the clone-versus-original difference of interest, depending solely on which speaker-verification encoder is used for measurement (43). For this reason, three independently trained encoders were used in the present study, and each clone score was contextualized against the patient’s own intra-speaker distribution and the cohort impostor distribution rather than reported as an isolated absolute value. Cohort-mean values of 0.933 with Resemblyzer, 0.969 with WavLM, and 0.806 with ECAPA-TDNN, each within 0.06 of the corresponding intra-speaker mean and well above the impostor mean, are consistent with the speaker-similarity ranges reported for the flow-matching F5-TTS architecture underlying this pipeline and with the large-scale Seed-TTS family of generation models. The cohort-level DTW-aligned MCD was 7.87 dB (range 4.21–11.86 dB). This finding indicates measurable spectral correspondence between the source and synthetic speech within the analysis framework used here. However, MCD is dependent on preprocessing, coefficient configuration, temporal alignment, and speech content; therefore, the absolute value should not be interpreted as a universal threshold of clinical or perceptual quality (44). Prosodic preservation is at least as clinically relevant as spectral fidelity, since mismatched intonation and pitch range are among the earliest cues by which listeners detect synthetic speech (45, 46). In the present cohort, mean fundamental frequency and its variability showed broad cohort-level correspondence between original and synthesized recordings, although individual deviations were observed. These findings support the feasibility of using clinic-grade smartphone recordings as the reference input for this workflow.

### Voice loss, identity, and clinical relevance

The literature documenting the impact of voice and communication loss in head and neck cancer is extensive and consistent in its conclusions (3, 47). Markers of social functioning track closely with depression and quality of life across head and neck cancer survivors, and qualitative work has consistently framed voice loss as a disruption of self-expression and personal identity rather than a purely functional impairment (48). Long-term cohort studies in tongue cancer specifically document substantial residual communication impairment at multi-year follow-up (49). Preservation of a patient’s own voice for use during the postoperative recovery period is therefore an identity-preserving intervention rather than merely a functional accommodation, and the outcome of primary relevance is whether patients and families accept the cloned voice as the patient’s own. The pattern observed on the Personalised Synthetic Voice Acceptance Questionnaire in this cohort is consistent with this framing. Both patient and family or attender ratings were highest on the domains of Identity-and-Ownership and Authenticity-and-Trust, approximately 4.5 in both groups, suggesting favourable perceived representation of the patient’s vocal identity within this exploratory questionnaire. Naturalness, Similarity, Emotional Acceptance, and Endorsement-and-Intention were rated more conservatively, and on these four domains the family or attender group consistently rated the cloned voice approximately 0.5 points higher than patients rated it themselves. Several explanations are possible, including differences in familiarity with one’s recorded voice and differences between self-perception and perception by an everyday listener. These explanations remain hypotheses and were not directly tested in the present study. Patient and family or attender ratings should therefore be regarded as complementary perspectives rather than measures with different levels of clinical relevance. The objective acoustic findings (**Tables 3**, **4**) collectively demonstrate preservation of speaker identity, spectral characteristics, and prosodic features. Occasional individual outliers were observed across speaker-verification encoders, reflecting the expected variability encountered in real-world recordings. Larger datasets will permit systematic investigation of patient-, recording-, and model-related factors associated with reduced speaker similarity.

### Implementation in the clinical pathway

Prehabilitation in head and neck oncology is an emerging paradigm with growing evidence of benefit across nutrition, swallowing, and speech domains (50). Preoperative voice banking is a natural extension within this framework, consistent with the broader principle of intervening before treatment, when functional outcomes can be most effectively influenced. The observed synthesis time was approximately 30 minutes per voice on the study workstation. Whether this processing time is compatible with routine same-day clinical delivery will depend on local workflow, staffing, and patient scheduling and was not evaluated in the present study. The technical maturity of zero-shot neural text-to-speech therefore supports further investigation of this approach within preoperative voice-banking workflows. The pipeline operates entirely offline, with no patient audio transmitted externally at any point, which substantially reduces the data-protection burden relative to cloud-based commercial voice-cloning services. Delivery may take the form of audio files provided on a portable storage device or a play-on-demand application on the patient’s own phone, a choice that represents a workflow decision rather than a technical constraint. Yamagishi and colleagues articulated, more than a decade ago, the long-term objective of speech-synthesis technologies for individuals with vocal disability, including voice banking and voice reconstruction. The technical maturity of zero-shot neural text-to-speech now makes this objective increasingly feasible for investigation within routine clinical workflows in multilingual South Asian settings (51).

### Data safety, consent, and governance

The property that renders zero-shot voice cloning clinically attractive, namely high speaker fidelity derived from a short reference clip, also constitutes a misuse risk should the cloned voice file or the underlying reference recording be exfiltrated (52, 53). In the present pipeline, no patient audio is transmitted to any external service at any stage; patient identifiers are removed at the point of file transfer; and the cloned voice file is retained on the institutional workstation. Written informed consent was obtained individually from every participant, including consent for voice recording, voice cloning, and research use of cloned outputs. Participants were informed that they could withdraw consent for future use of their recorded or cloned voice material. Broader institutional governance procedures concerning long-term storage, access, deletion, and export of synthetic voice assets were not yet formally established and are identified as an area requiring further development. The governance questions raised by artificial-intelligence methods in medical research more broadly are not unique to voice synthesis and have been addressed in the wider literature (54).

### Limitations

This is a single-centre study comprising 26 patients, which limits generalizability. The questionnaire ratings reported here are pooled across postoperative time-points and represent self-report at a single follow-up; longitudinal within-patient trajectories and within-patient pre- versus postoperative acoustic comparison have not yet been performed. The cohort comprised oral cavity cancers exclusively, and these findings do not generalize to laryngeal cancer, in which the pre-treatment voice profile differs materially. The Personalised Synthetic Voice Acceptance Questionnaire was intentionally developed as an exploratory feasibility instrument for this pilot study and has not undergone formal psychometric validation. Evaluation of internal consistency, construct validity, factor structure, and test-retest reliability will require substantially larger cohorts and is planned as the next stage of this programme. Reference transcript preparation differed slightly across languages, with English transcripts generated using Whisper and clinician verification, whereas Indian-language transcripts were prepared directly by clinicians. Although verification was performed for all transcripts, minor language-specific variability cannot be excluded. The two synthesis outputs had different evidentiary roles. Output B used the participant’s own transcript and therefore represents a content-matched comparison that may favour similarity estimates, whereas Output A provides a more relevant assessment of performance on content not present in the recorded speech. Future studies should incorporate systematically held-out speech material.

Training and synthesis were performed on a single, fixed laptop configuration; the pipeline has not been validated on cloud infrastructure or mobile devices, has not been tested for real-time conversational use, and requires approximately 30 minutes per voice on this configuration. The cloned voice retains the prosodic characteristics of the recorded speech and does not yet reproduce the full range of emotional expression; therefore, its performance for functional postoperative communication remains to be evaluated, particularly during spontaneous rather than scripted speech. Although four languages were represented, Tamil and Hindi were represented by only two and one participants, respectively. Consequently, language-specific subgroup analyses were not performed because such comparisons would be statistically underpowered and potentially misleading.

An additional limitation is that 8 of 34 enrolled participants (23.5%) were excluded because their recordings were judged unsuitable as reference material. This represents an important component of the practical feasibility of the workflow and may introduce selection effects if patients unable to provide technically adequate recordings differ systematically from those who can.

The present study evaluated acceptance of synthetic voice generation rather than postoperative communication disability or quality of life. Accordingly, validated PROMs such as the Voice Handicap Index (VHI), Voice-Related Quality of Life (V-RQOL), Speech Handicap Index (SHI), and the EORTC QLQ-H&N35 were not incorporated into this feasibility study. Future studies should examine the relationship between PSV-AQ scores and established voice- and speech-related PROMs to establish convergent validity and determine whether acceptance of a cloned voice translates into measurable improvements in communication-related quality of life.

Because participants were aware that the intervention formed part of their treating institution’s research programme, social desirability bias cannot be completely excluded despite efforts to administer questionnaires in a standardized manner.

Real-world postoperative deployment was beyond the scope of the current study.

### Future directions

Three extensions of this work are immediate. First, completion of longitudinal questionnaire data collection will permit direct testing of the alignment between objective acoustic similarity and subjective acceptance across the six acceptance domains and over time. Second, extension of the cohort to laryngeal cancer and laryngectomy patients will determine whether the same pipeline transfers to a population whose pre-treatment voice is itself frequently abnormal. Third, expansion of language coverage beyond the four languages represented in this cohort, together with evaluation of the pipeline against spontaneous rather than read-aloud speech, will help define the limits of this architecture in clinical use. A multi-centre extension and formal psychometric validation of the questionnaire represent the longer-term objectives of this programme of work. Future studies with larger cohorts should examine the relationship between objective acoustic fidelity measures and subjective acceptance scores using correlation and multivariable modelling to determine which acoustic characteristics most strongly influence perceived voice authenticity. Although standardized reading passages remain the conventional stimulus for acoustic voice analysis because they reduce phonetic variability, future work should also extend evaluation to spontaneous conversational speech, emotionally expressive speech, and frequently used personalized phrases, which may better reflect the intended real-world use of cloned voices in postoperative communication.

### Conclusion

Zero-shot neural text-to-speech can generate personalised synthetic voices from brief preoperative clinic-grade recordings in patients undergoing major glossectomy. In this single-centre feasibility cohort, the generated voices demonstrated measurable speaker similarity and acoustic correspondence across multiple encoders, with preliminary evidence of perceived identity and acceptance among patients and family or attender listeners. These findings support further prospective evaluation of personalised synthetic voices as potential assistive communication tools after major glossectomy. However, this study demonstrates technical feasibility and preliminary acceptance only; postoperative communication performance, intelligibility, quality of life, and clinical benefit were not evaluated and remain to be established in prospective clinical studies.

## Data Availability

The datasets presented in this article are not readily available because Individual voice data is not available due to privacy and ethical issues. Requests to access the datasets should be directed to karthik.nag.rao@gmail.com.

## Supplementary material

Supplementary File 1 (Passages.docx): standardized reading passages in Kannada, Hindi, Tamil, and Indian English used to generate Output A.

Supplementary File 2 (PSV-AQperpatientratings.xlsx): per-patient PSV-AQ scores for all 26 included patients, three sheets, Patientselfreport, Familyattender, and Summary.
