# Supplementary material for: The tongue may not move, but the voice will: preoperative AI voice cloning for identity preservation in major glossectomy — a prospective feasibility study
Source: Front Oncol. 2026 Sep 21;16:1915964. doi: 10.3389/fonc.2026.1915964 (PMC13634861; doi:10.3389/fonc.2026.1915964)
Supplement: Supplementary file 1 [file Table1.docx]

# Supplementary File 1. Standardised reading passages used for Output A

The Output A clone for each patient was generated from a 90–100-word

city-themed reading passage, translated into the patient's preferred

vernacular. The four parallel translations are reproduced below.

## Kannada

ಬೆಂಗಳೂರು ಕರ್ನಾಟಕ ರಾಜ್ಯದ ರಾಜಧಾನಿ ಮತ್ತು ಅತಿ ದೊಡ್ಡ ನಗರವಾಗಿದೆ. ಇದನ್ನು ಗಾರ್ಡನ್ ಸಿಟಿ

ಹಾಗೂ ಭಾರತದ ಸಿಲಿಕಾನ್ ವ್ಯಾಲಿ ಎಂದು ಕರೆಯಲಾಗುತ್ತದೆ. ಬೆಂಗಳೂರು ತನ್ನ ಹಸಿರು ವಾತಾವರಣ,

ಸುಂದರ ಉದ್ಯಾನಗಳು ಮತ್ತು ಆಧುನಿಕ ತಂತ್ರಜ್ಞಾನ ಸಂಸ್ಥೆಗಳಿಗಾಗಿ ಪ್ರಸಿದ್ಧವಾಗಿದೆ. ನಗರದ ಪ್ರಮುಖ

ಆಕರ್ಷಣೆಗಳಲ್ಲಿ ಲಾಲ್‌ಬಾಗ್, ಕಬ್ಬನ್ ಪಾರ್ಕ್ ಮತ್ತು ಬೆಂಗಳೂರು ಅರಮನೆ ಸೇರಿವೆ. ಬೆಂಗಳೂರಿನಲ್ಲಿ

ವರ್ಷಪೂರ್ತಿ ಸಮಶೀತೋಷ್ಣ ಹವಾಮಾನವಿರುವುದರಿಂದ ಇದು ವಾಸಿಸಲು ಅನುಕೂಲಕರ ನಗರವಾಗಿದೆ.

## Hindi

बेंगलुरु कर्नाटक राज्य की राजधानी और सबसे बड़ा शहर है। इसे गार्डन सिटी और भारत

की सिलिकॉन वैली के नाम से जाना जाता है। बेंगलुरु अपने हरियाली भरे वातावरण,

सुंदर बगीचों और आधुनिक प्रौद्योगिकी संस्थानों के लिए प्रसिद्ध है। शहर के प्रमुख

आकर्षणों में लालबाग, कब्बन पार्क और बेंगलुरु पैलेस शामिल हैं। बेंगलुरु में साल

भर समशीतोष्ण जलवायु रहती है, जिससे यहाँ रहना सुखद हो जाता है।

## Tamil

பெங்களூரு கர்நாடக மாநிலத்தின் தலைநகரும் மிகப் பெரிய நகரமும் ஆகும். இதை கார்டன்

சிட்டி என்றும் இந்தியாவின் சிலிக்கான் வேலி என்றும் அழைக்கின்றனர். பெங்களூரு

தனது பசுமை சூழல், அழகான பூங்காக்கள் மற்றும் நவீன தொழில்நுட்ப நிறுவனங்களுக்கு

பெயர் பெற்றது. நகரத்தின் முக்கிய இடங்களில் லால்பாக், கப்பன் பார்க் மற்றும்

பெங்களூரு அரண்மனை ஆகியவை அடங்கும். பெங்களூரில் ஆண்டு முழுவதும் நிலையான

குளிர்ச்சியான வானிலை இருப்பதால் இது வாழ்வதற்கு வசதியான நகராக உள்ளது.

## English (rendered into the IndicF5 pipeline via Devanagari phonetic transliteration)

Bangalore is the capital and largest city of the state of Karnataka. It is

known as the Garden City and the Silicon Valley of India. Bangalore is famous

for its green environment, beautiful parks and modern technology institutions.

The major attractions of the city include Lalbagh, Cubbon Park and Bangalore

Palace. Bangalore has a temperate climate all year round, making it a pleasant

city to live in.
